# Morphologic and molecular characterization of new Cyclospora species from Ethiopian monkeys: C. cercopitheci sp.n., C. colobi sp.n., and C. papionis sp.n.

**DOI:** 10.3201/eid0505.990506

**Published:** 1999

**Authors:** M L Eberhard, A J da Silva, B G Lilley, N J Pieniazek

**Affiliations:** Division of Parasitic Diseases, Centers for Disease Control and Prevention, Atlanta, Georgia 30341-3724, USA. mle1@cdc.gov

## Abstract

In recent years, human cyclosporiasis has emerged as an important infection, with large outbreaks in the United States and Canada. Understanding the biology and epidemiology of Cyclospora has been difficult and slow and has been complicated by not knowing the pathogen s origins, animal reservoirs (if any), and relationship to other coccidian parasites. This report provides morphologic and molecular characterization of three parasites isolated from primates and names each isolate: Cyclospora cercopitheci sp.n. for a species recovered from green monkeys, C. colobi sp.n. for a parasite from colobus monkeys, and C. papionis sp.n. for a species infecting baboons. These species, plus C. cayetanensis, which infects humans, increase to four the recognized species of Cyclospora infecting primates. These four species group homogeneously as a single branch intermediate between avian and mammalian Eimeria. Results of our analysis contribute toward clarification of the taxonomic position of Cyclospora and its relationship to other coccidian parasites.


### Morphologic and Molecular Characterization of New *Cyclospora* Species from Ethiopian Monkeys: *C. cercopitheci* sp.n., *C. colobi* sp.n., and *C. papionis* sp.n.

**Mark L. Eberhard,* Alexandre J. da Silva,* Bruce G. Lilley,† and Norman J. Pieniazek***
*Centers for Disease Control and Prevention, Atlanta, Georgia, USA; and
†University of Alabama at Birmingham, Birmingham, Alabama, USA

In recent years, human cyclosporiasis has emerged as an important infection, with large outbreaks in the United States and Canada. Understanding the biology and epidemiology of *Cyclospora* has been difficult and slow and has been complicated by not knowing the pathogen's origins, animal reservoirs (if any), and relationship to other coccidian parasites. This report provides morphologic and molecular characterization of three parasites isolated from primates and names each isolate: *Cyclospora cercopitheci* sp.n. for a species recovered from green monkeys, *C. colobi* sp.n. for a parasite from colobus monkeys, and *C. papionis* sp.n. for a species infecting baboons. These species, plus *C. cayetanensis,* which infects humans, increase to four the recognized species of *Cyclospora* infecting primates. These four species group homogeneously as a single branch intermediate between avian and mammalian *Eimeria*. Results of our analysis contribute toward clarification of the taxonomic position of *Cyclospora* and its relationship to other coccidian parasites.

*Cyclospora cayetanensis,* a coccidian parasite recently described as a human pathogen causing prolonged watery diarrhea (1), has been identified as the cause of large, multistate outbreaks of diarrhea in the United States associated with imported produce, most notably raspberries (2,3). Molecular phylogenetic analysis showed that *Cyclospora* is closely related to *Eimeria* species (4), especially to mammalian *Eimeria* species (5). The parasite has been reported from many geographic regions but seems to be endemic in tropical countries. Recent foodborne outbreaks in the United States and Canada have generated considerable scientific interest and numerous questions about this organism; one of the most perplexing has to do with the possible role of other animals in harboring the infection and serving as a source of contamination.

Address for correspondence: Mark L. Eberhard, Division of Parasitic Diseases, Mail Stop F13, Centers for Disease Control and Prevention, 4770 Buford Hwy, NE, Atlanta, GA 30341-3724, USA; fax: 770-488-4253; e-mail: mle1@cdc.gov.

In 1996, Smith and colleagues reported the presence of *C. cayetanensis*-like oocysts in the feces of 37 of 37 baboons and 1 of 15 chimpanzees examined from the Gombe National Park, Tanzania. Other reports have documented *C. cayetanensis*-like oocysts in fecal samples from chickens in Mexico (6), a duck in Peru (7), and dogs in Brazil (8). However, only the Smith report (9) suggests a true natural host.

During spring 1997, we collected stool samples from free-ranging baboons (*Papio anubis*) and colobus monkeys (*Colobus guereza*) in Wollega Province in western Ethiopia. A high percentage of samples were positive for *Cyclospora* oocysts, but the organism, including sporulated oocysts, could not be completely described because the samples were fixed in formalin. In spring 1998, we returned to Wollega Province, collected additional stool samples from three species of primates (baboons, colobus, and African green monkeys [*Cercopithecus aethiops*]), and placed these samples in potassium dichromate for subsequent biologic and molecular studies. This report describes the results of those collections, provides molecular phylogenetic analysis, and names the newly identified parasites.

### The Study

We collected stool samples from troops of baboons and green monkeys by following them as they foraged and from colobus monkeys by quietly waiting under trees in which monkeys were sitting. Only fresh stool samples were collected, and in neither situation was it possible to determine the age or sex of the animal that produced the sample. On several occasions, samples from more than one animal of the same species were pooled; these are referred to as composite samples.

In the collections from 1997, each stool sample was placed directly in 10% formalin. In the collections from 1998, all stool samples were suspended in water and allowed to settle. The sediment was sieved and resuspended in clean water. The resulting sediment was mixed with a 2.5% aqueous (w/v) potassium dichromate ($K_2Cr_2O_7$) solution in a 3:1 ratio and allowed to settle. The supernatant was discarded, and fresh potassium dichromate solution was added in a 3:1 ratio. The potassium dichromate-stool mixture was kept at room temperature in 50-ml conical centrifuge tubes and returned to Atlanta.

The *C. cayetanensis* oocysts used in comparative studies were collected from stools from a 1997 Florida outbreak linked to consumption of Guatemalan raspberries and from stools collected in Leogane, Haiti.

### DNA Extraction

DNA was extracted from 500-µl aliquots of stool samples, following the protocol of da Silva et al. (10). Extracted DNA was stored at 4°C until polymerase chain reaction (PCR) amplification was performed on the small subunit ribosomal RNA (SSU-rRNA) coding region of the genome. Both strands of PCR products were sequenced directly by using an automated DNA sequencer. We used a nested PCR protocol with primers CYCF1E and CYCR2B for the first step of the amplification and primers CYCF3E and CYCR4B for the second (11).

### Results

Examination of stools collected in 1997 showed that 15 (68%) of 22 baboons and 9 (60%) of 15 colobus monkeys had detectable *Cyclospora* infections. In individual stool samples collected in 1998, 10 (50%) of 20 baboons, 0 of 11 colobus monkeys, and 1 (6%) of 16 green monkeys had detectable infections with *Cyclospora*. In composite stool samples collected in 1998, 2 (100%) of 2 baboon, 1 (50%) of 2 colobus monkey, and 0 of 3 green monkey samples tested positive for *Cyclospora*.

### Sequencing of the SSU-rRNA Coding Region and Phylogenetic Analysis

SSU-rRNA sequences amplified from the *C. cayetanensis* isolates from Haiti and Florida were identical and showed seven differences from the sequence described by Relman et al. (4). Three of these differences correct previously unresolved bases: A at positions 400 and 549, and G at position 1694. Two other differences most probably correct a sequencing error, as they constitute an inversion next to an unresolved position (T at position 1695 and G at position 1696). The significance of the two remaining differences at positions 696 and 1360 is unknown at this time. The new sequence for *C. cayetanensis* SSU-rRNA coding region was deposited in GenBank and assigned accession number AF111183 (Table).

SSU-rRNA sequences obtained for two baboon isolates were identical. The colobus and baboon *Cyclospora* isolates were assigned GenBank accession numbers AF111186 and AF111187, respectively. Sequencing of SSU-rRNA coding region of *C. cercopitheci* from a single African green monkey specimen showed a heterozygotic position, T/A at position #280. This may reflect mixed infection with two closely related isolates or may represent polymorphism among several copies of this gene in a single isolate. Thus, both green monkey SSU-rRNA sequences were submitted separately to GenBank and were assigned accession numbers AF111184 for the green monkey *Cyclospora* sequence #1 and AF111185 for sequence #2 (Table).

The phylogenetic trees generated by the PUZZLE and PAUP programs displayed similar topologic features and demonstrated that on the basis of the SSU-rRNA the *Cyclospora* isolates from monkeys are distinct from each other and from *C. cayetanensis* of humans (Figure 1). The sequence identities between the human isolate with the baboon, colobus monkey, and green monkey isolates 1 and 2 were 98.6%, 98.7%, and

Table. New *Cyclospora* species from Ethiopian monkeys

| Characteristics | *Cyclospora cercopitheci* | *C. colobi* | *C. papionis* |
|---|---|---|---|
| Host | *Cercopithecus aethiops* Linnaeus, 1758, African green or vervet monkey | *Colobus guereza* Ruppell, 1835, colobus monkey | *Papio anubis* Lesson, 1827, olive baboon |
| Oocysts | Spherical; 8 - 10 µm (mean 9.2) in diameter. Outer wall smooth. Wall autofluoresces in UV wavelength. | Small, spherical, 8 - 9 µm (mean 8.3) in diameter. Outer wall smooth. Wall autofluoresces in UV wavelength. | Spherical, 8 - 10 µm (mean 8.8) in diameter. Outer wall smooth. Wall autofluoresces in UV wavelength. |
| Sporocysts | Two per mature oocyst Lemon-shaped, 6-7 by 4-5 µm, with L/W ratio 1.5 | Two per mature oocyst Lemon-shaped, 7-8 by 4-5 µm, with L/W ratio 1.66 | Two per mature oocyst Lemon-shaped, 7-8 by 4-5 µm, with L/W ratio ratio 1.66 |
| Stieda bodies | A prominent stieda body present; sub- and parastieda bodies absent | A prominent stieda body present; sub- and parastieda bodies absent | A prominent stieda body present; sub- and parastieda bodies absent |
| Sporocyst residuum | Prominent; made up of clumped globules | Prominent, irregularly shaped; 2-4 µm in diameter | Prominent, irregularly shaped; 2-3 by 3-4 µm in diameter |
| Micropyle | Absent | Absent | Absent |
| Sporozoites | Two per sporocyst 10-13 by 1.5 µm; tapered at both ends | Two per sporocyst 10-13 by 2 µm; tapered at both ends | Two per sporocyst 10-13 by 1.5 µm; tapered at both ends |
| Remarks | Marginally larger than other two species Heterozygotic position, T or A at position #280; therefore, SSU-rRNA sequences submitted separately. Assigned accession nos. AF111184 and AF111185 | Marginally smaller than the two other species Sequence of SSU-rRNA assigned accession no. AF111186. Poorest sporulation rate of three species | The most commonly encountered of the three species. Sequence of SSU-rRNA assigned accession no. AF111187 |

98.4%, respectively. The phylogenetic relationship observed between *Cyclospora* and *Eimeria* species confirmed previous findings (4,5) with three distinct clades: avian *Eimeria*, mammalian *Eimeria*, and *Cyclospora*.

### Conclusions

The genus *Cyclospora* was formed by Schneider in 1881 for organisms recovered from myriapods (terrestrial arthropods in the subphylum Mandibulata, Class Diplopoda [millipedes] and Class Chilopoda [centipedes]). Most knowledge about the genus *Cyclospora* is based on recently recognized species described from insectivores (moles) (12), heteromyid rodents in the southwestern United States (13), and humans (1).

In 1994, Ortega and colleagues described *C. cayetanensis* from human fecal material in Peru. In 1997, they described the parasite's intracellular life cycle in the duodenum and jejunum (14). *C. cayetanensis* differs significantly from all other described species not only in its host but also in its oocyst stage, which is much smaller and spherical rather than oblong. The recovery from nonhuman primates of other species of *Cyclospora* that produce small, spherical oocysts seems to suggest two distinct groupings: species that infect insectivores and rodents and produce large, oblong oocysts and those that infect primates (including humans) and produce small, spherical oocysts.

The geographic and host range for *C. papionis*, *C. colobi*, and *C. cercopitheci* needs to be defined. These primate species of *Cyclospora* are easily distinguished at the molecular level, but not at the light-microscope level. That *C. papionis*,

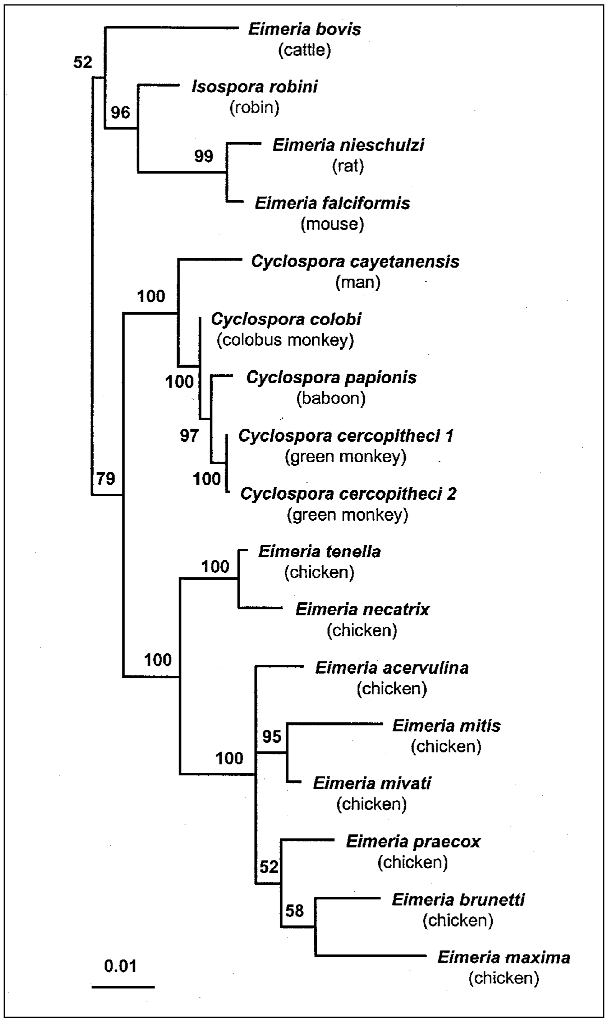

*C. colobi*, and *C. cercopitheci* are species distinct from each other and from *C. cayetanensis* of humans is well substantiated, considering the molecular phylogeny based on the SSU-rRNA sequence data. This assumes that the differences in SSU-RNA reflect distinct species. For the time being, this method is probably the best for defining morphologically similar species. The separation of these parasite species is further supported by the distinct separation of the primate species on biologic grounds. Baboons are omnivorous, spend most of their time on the ground foraging for food, and have relatively large home ranges. Green monkeys spend a greater amount of time in trees but do forage on the ground. The canopy-dwelling colobus monkeys, on the other hand, have the smallest home range and are strict herbivores, predominantly eating leaves of certain trees.

The *Cyclospora* observed in baboons from Tanzania (9) is likely the same species as *C. papionis* from Ethiopia. A high percentage of baboons in the Gombe Stream Preserve are infected with a *Cyclospora* species indistinguishable from *C. papionis* (pers. obs.; Whittier, pers. comm.). Moreover, three sequences from Gombe baboon isolates submitted recently to GenBank (15) show only one base change from our sequence with each isolate (C to T at #1360 with

Figure 1.[1] Phylogenetic tree for small subunit ribosomal RNA sequences of *Cyclospora* and *Eimeria* species. Quartet puzzling maximum likelihood results are shown, with *Toxoplasma gondii* as the outgroup. After analysis, the outgroup branch was removed for clarity. Numbers to the left of the nodes indicate the quartet puzzling support for each internal branch. The scale bar indicates an evolutionary distance of 0.01 nucleotides per position in the sequence. Vertical distances are for clarity only. GenBank accession numbers of the sequences used for analysis: *Cyclospora cayetanensis,* AF111183; *C. cercopitheci* 1, AF111184; *C. cercopitheci* 2, AF111185; *C. colobi,* AF111186; *C. papionis,* AF111187; *Eimeria acervulina,* U67115; *E. bovis,* U77084; *E. brunetti,* U67116; *E. falciformis,* AF080614; *E. maxima,* U67117; *E. mitis,* U40262; *E. mivati,* U76748; *E. necatrix,* U67119; *E. nieschulzi,* U40263; *E. praecox,* U67120; *E. tenella,* U40264; *Isospora robini,* AF080612; and *Toxoplasma gondii,* U12138. The sequences were aligned with the program CLUSTALW (20). Phylogenetic analysis was done with the maximum likelihood method-based PUZZLE program (21), as well as with the parsimony method-based PAUP program (22). Unreliably aligned regions were removed, and the final length of the alignment was 1692 columns. Aligned sequences are available from the authors upon request.

[1]Initially, we used a nested PCR protocol using primers CYCF1E and CYCR2B for the first step of the amplification and primers CYCF3E and CYCR4B for the second step (11). Samples were also amplified by using sets of PCR primers designed on the basis of the primers described above, but with the restriction sites removed. The primer CYCF1 (5'-ATTACCCAATGAA AACAGTTT- 3') was used in pairs with the primer CYCR4 (5'-TCGTCTTCAAACCCCCTACTG-3') to generate a DNA fragment of 577 bp. The other pair of primers, CYCF3 (5'-GCCTTCCGCGCTTCGCTGCGT-3') and CYCR2 (5'-TGC AGGAGAAGCCAAGGTAGG-3') was used to generate a fragment of 283 bp. To generate fragments spanning the full length of the SSU-rRNA coding region, we used generic apicomplexan PCR primer CRYPTOF (5'-AACCTG GTTGATCCTGCCAGT-3'), specific for the 5' end of the SSU-rRNA molecule and the apicomplexan generic PCR primer CRYPTOR (5'-GCTTGATCCTTCTGCAGGTTCACC TAC-3'), specific for the 3' end of this molecule. These generic primers were combined with the *Cyclospora*-specific primers (CYC-series, see above) to amplify overlapping fragments spanning the whole SSU-rRNA molecule. PCR products were analyzed by electrophoresis on 2% SeaKem GTG agarose (Cat. No. 50074, FMC Bioproducts, Rockland, ME), stained with ethidium bromide and visualized on a UV transilluminator.

sequences AF065566 and AF065567; C to T at #184 with AF065568), if unresolved base positions in their sequences are disregarded (three positions in AF065566 and AF065567 and six positions in AF065568).

The topology of the tree (Figure 1) displays the distinct *Cyclospora* species as a monophyletic branch with phylogenetic proximity to the genus *Eimeria*. The proximity between these coccidian genera has been demonstrated (4,5); we included in the tree an additional SSU-rRNA sequence of *E. falciformis*, a parasite of mice. The addition of this species clarified the resolution of the tree into three distinct clades: mammalian *Eimeria*, avian *Eimeria*, and *Cyclospora*. With the addition of molecular data for more species, especially the species of *Cyclospora* described from mammals other than primates, it may be reasonable to consider reclassifying the *Cyclospora* of primates (including humans) and either the bird or mammalian *Eimeria* to a new genus. However, morphologic and molecular taxonomists continue to struggle with the relationships within the coccidia. Morphologic criteria for naming the genera have provided a stable basis for many years. On the other hand, molecular data, based on the genetic information of these same organisms, suggest affiliations that do not always coincide with the existing associations based on morphologic features. Sterling and Ortega (16) suggest that small subunit rRNA sequences of *Isospora* should be compared with those of *Cyclospora* to help clarify taxonomic issues. They also point out the role of molecular taxonomy in establishing the validity of species and taxonomic groupings. Carreno and Barta (17) provided sequencing data for several species of *Isospora* and demonstrated the phylogenetic separation of various clades of *Isospora,* both with and without Stieda bodies. They propose separating mammalian species with Stieda bodies into the family Eimeriidae and retaining those without Stieda bodies in the family Sarcocystidae. We included sequences of *I. robini* in our analysis, and *Cyclospora* remains as a clearly separate grouping.

On the basis of the topology of the tree and the distance values obtained, the simian isolates are more closely related to each other than to *C. cayetanensis* of humans. This undoubtedly reflects host differences as well as other biologic features of each species. However, further molecular studies are needed to demonstrate whether these *Cyclospora* species described from lower primates occur in humans, or conversely, whether *C. cayetanensis* can occur in monkeys. At least in East Africa, researchers should continue to evaluate material collected from humans and nonhuman primates with care. We are continuing our efforts to determine whether other primate species are infected with these or distinct species of *Cyclospora*. Studies of human isolates of *C. cayetanensis* from different geographic regions have, thus far, not demonstrated any molecular differences. This further substantiates the taxonomic significance of the molecular differences detected between the *Cyclospora* from humans and lower primates.

### Appendix I

### Stool Processing Procedures

Stool samples collected in 1997 were processed by a conventional formalin-ethyl acetate sedimentation concentration procedure. A portion of the sediment was examined by UV fluorescent microscopy (18). Some positive samples were also stained by the acid-fast or hot safranin techniques (19). For stools collected in 1998, an aliquot of each sample was washed because potassium dichromate suppresses the autofluorescence of the oocysts. Any oocysts observed in the samples examined from the collection of 1998 were graded as either sporulated or unsporulated. Part of the remaining specimen in potassium dichromate was processed over sucrose gradient to harvest oocysts. Purified oocysts were returned to clean 2.5% potassium dichromate solution for storage, and portions of the purified oocysts were used for morphologic studies.

To excyst sporocysts and sporozoites, one of two procedures was used. If the intent was to obtain free sporocysts, but not sporozoites, a small drop of solution containing oocysts was placed on a glass slide and covered. To induce rupture of the oocyst wall, the coverslip was tapped with a blunt glass rod and then rotated on the slide. To obtain free sporozoites, one of two excysting fluids were used: either a solution made up in DMEM containing 0.25% trypsin plus 0.75% sodium taurocholate or a solution made up in PBS containing 0.25% trypsin, 0.75% sodium taurocholate, and 20 mM cystine HCl. Both solutions worked equally well. The oocysts were incubated in the excysting fluid for 2 hours in a heat block at 37°C.

### Appendix II

### Taxonomic Description of the Parasites

***Cyclospora cercopitheci*** **sp.n.** (Figures 2–3, 9)
**Type host:** *Cercopithecus aethiops* Linnaeus, 1758, African green or vervet monkey.
**Type locality:** Gimbie, Wollega Province, Ethiopia.
**Prevalence:** found in 6% of green monkeys sampled.
**Site of infection:** Unknown, oocysts collected from feces.
**Material deposited:** Phototypes and syntypes, U.S. National Parasite Collection, accession number 088837.
**Etymology:** The species name was derived from the genus name for the primate host from which this parasite was recovered.
**Remarks:** Sequencing of SSU-rRNA coding region of *C. cercopitheci* from a single African green monkey specimen revealed that there was a heterozygotic position, T or A at position #280. Thus, SSU-rRNA sequences for these two isolates were submitted separately to GenBank and were assigned accession numbers AF111184 for *C. cercopitheci* sequence #1 and AF111185 for *C. cercopitheci* sequence #2.

***Cyclospora colobi*** **sp.n.** (Figures 4–5, 10)
**Type host:** *Colobus guereza* Ruppell, 1835, colobus monkey.
**Type locality:** Gimbie, Wollega Province, Ethiopia.
**Prevalence:** Up to 60% of colobus monkeys sampled.
**Site of infection:** Unknown, oocysts collected from feces.
**Material deposited:** Phototypes and syntypes, U.S. National Parasite Collection, accession number 088838.
**Etymology:** The species name was derived from the genus name of the primate host from which this parasite was recovered.
**Remarks:** This species is marginally smaller than the two other species described from monkeys, but the overlap in sizes between the species does not allow a clear distinction on the basis of size. Sporulation of material collected from colobus monkeys was poor in comparison with *C. papionis* from baboons, despite the fact that material was collected and handled in a similar fashion. Sequence of the SSU-rRNA coding region for this species was deposited in GenBank and was assigned accession number AF111186.

***Cyclospora papionis*** **sp.n.** (Figures 6–9, 11)
**Type host:** *Papio anubis* Lesson, 1827, olive baboon.
**Type locality:** Gimbie, Wollega Province, Ethiopia.
**Prevalence:** Found in >50% of baboons sampled.
**Site of infection:** Unknown, oocysts collected from feces.
**Material deposited:** Phototypes and syntypes, U.S. National Parasite Collection, accession number 088839.
**Etymology:** The species name was derived from the genus name for the primate host from which this parasite was recovered.
**Remarks:** More than 90% of the oocysts collected from baboons underwent sporulation in virtually all of the positive samples. Sequence of the SSU-rRNA coding region for this species was deposited in GenBank and was assigned accession number AF111187.

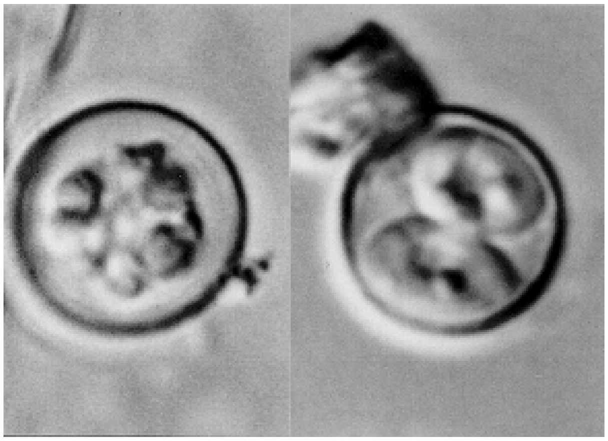

Figures 2–3. Photomicrographs of *Cyclospora cercopitheci* sp. n. from feces of African green monkeys (*Cercopithecus aethiops*) in Ethiopia, Africa. x 3300. 2. Unsporulated oocyst from feces. 3. Sporulated oocyst after 1 month of incubation.

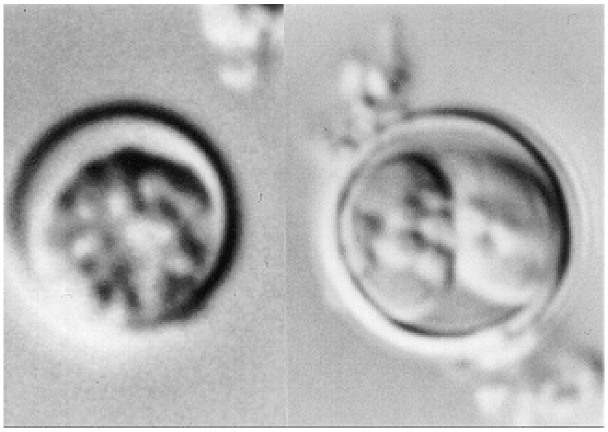

Figures 4–5. Photomicrographs of *Cyclospora colobi* sp. n. from feces of colobus monkeys (*Colobus guereza*) in Ethiopia, Africa. x 3300. 4. Unsporulated oocysts from feces. 5. Sporulated oocyst after 1 month of incubation.

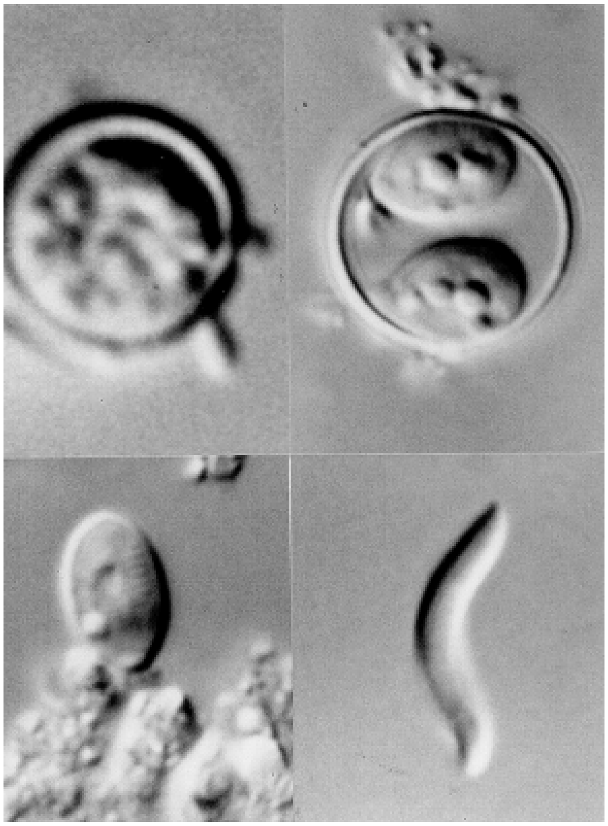

Figures 6–9. Photomicrographs of *Cyclospora papionis* sp. n. oocysts from feces of baboons (*Papio anubis*) in Ethiopia, Africa. x 3300. 6. Unsporulated oocysts from feces. 7. Sporulated oocyst after 1 month of incubation. 8. Free sporocyst from ruptured oocyst. 9. Free sporozoite from ruptured sporocyst.

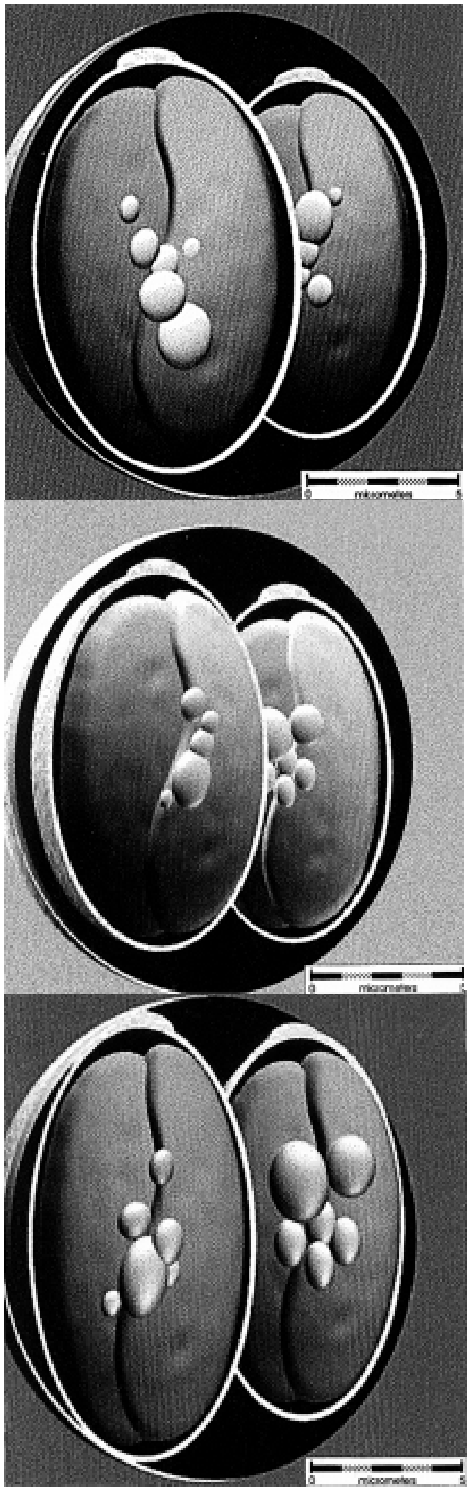

Figures 10–12. Line drawings of sporulated oocysts of *Cyclospora* from feces of primates in Ethiopia, Africa. Bar = 5 µm. 10. *Cyclospora cercopitheci* sp. n. from African green monkeys, *Cercopithecus aethiops*. 11. *Cyclospora colobi* sp. n. from colobus monkeys, *Colobus guereza*. 12. *Cyclospora papionis* sp. n. from baboons, *Papio anubis*.

### Acknowledgments

The authors thank Oleg Ditrich for providing useful hints on the Latin grammar usage and Robert Hobbs, Jr., for the computer images of the oocyst composite drawings of each species.

Dr. Eberhard is head of the Biology and Diagnostics Branch, Division of Parasitic Diseases, National Center for Infectious Diseases, CDC. Trained in classical parasitology, he has broad interests in the diagnosis and biology of parasitic infections. His research interests include the identification of unusual parasites and zoonotic infections.

### References

1. Ortega YR, Gilman RH, Sterling CR. A new coccidian parasite (Apicomplexa: Eimeriidae) from humans. J Parasitol 1994;80:625-9.
2. Koumans EH, Katz DJ, Malecki JM, Kumar S, Wahlquist SP, Arrowood MJ, et al. An outbreak of cyclosporiasis in Florida in 1995: a harbinger of multistate outbreaks in 1996 and 1997. Am J Trop Med Hyg 1998;59:235-42.
3. Herwaldt BL, Ackers ML, the Cyclospora Working Group. An outbreak in 1996 of cyclosporiasis associated with imported raspberries. N Engl J Med 1997;336:1548-56.
4. Relman DA, Schmidt TM, Gajadhar A, Sogin M, Cross J, Yoder K, et al. Molecular phylogenetic analysis of *Cyclospora*, the human intestinal pathogen, suggests that it is closely related to *Eimeria* species. J Infect Dis 1996;173:440-5.
5. Pieniazek NJ, Herwaldt BL. Reevaluating the molecular taxonomy: is human-associated *Cyclospora* a mammalian *Eimeria* species? Emerg Infect Dis 1997;3:381-3.
6. García-López HL, Rodríguez-Tovar LE, Medina de la Garza CE. Identification of *Cyclospora* in poultry. Emerg Infect Dis 1996;2:356-7.
7. Zerpa R, Uchima N, Huicho L. *Cyclospora cayetanensis* associated with watery diarrhoea in Peruvian patients. Am J Trop Med Hyg 1995;98:325-9.
8. Yai LE, Bauab AR, Hirschfeld MP, de Oliveira ML, Damaceno JT. The first two cases of *Cyclospora* in dogs, Sao Paulo, Brazil. Rev Inst Med Trop Sao Paulo 1997;39:177-9.
9. Smith HV, Paton CA, Girdwood RWA, Mtambo MMA. *Cyclospora* in non-human primates. Vet Rec 1996;138:528.
10. da Silva AJ, Bornay-Llinares FJ, Moura INS, Slemenda SB, Tuttle JL, Pieniazek NJ. Fast and reliable extraction of protozoan parasite DNA from fecal specimens. Molecular Diagnosis 1999;4:57-64.
11. Pieniazek NJ, Slemenda SB, da Silva AJ, Alfano EM, Arrowood AJ. PCR confirmation of infections with *Cyclospora cayetanensis*. Emerg Infect Dis 1996;2:357-9.
12. Ford PL, Duszynski DW, McAllister CT. Coccidia (Apicomplexa) from heteromyid rodents in the southwestern United States, Baja California, and northern Mexico with three new species from *Chaetodipus hispidus*. J Parasitol 1990;76:325-31.
13. Ford PL, Duszynski DW. Coccidian parasites (Apicomplexa: Eimeriidae) from insectivores. VII. Six new species from the hairy-tailed mole, *Parascalops breweri*. J Parasitol 1989;75:508-13.
14. Ortega YR, Nagle R, Gilman RH, Watanabe J, Miyagui J, Quispe H, et al. Pathologic and clinical findings in patients with cyclosporiasis and a description of intracellular parasite life-cycle stages. J Infect Dis 1997;176:1584-9.
15. Lopez FA, Manglicmot JS, Schmidt TM, Yeh C, Smith HV, Relman DA. Molecular organization of *Cyclospora*-like organisms from baboons. J Infect Dis 1999;179:670-6.
16. Sterling CR, Ortega YR. *Cyclospora*: an enigma worth unraveling. Emerg Infect Dis 1999;5:48-53.
17. Carreno RA, Barta JR. An eimeriid origin of isosporoid coccidia with stieda bodies as shown by phylogenetic analysis of small subunit ribosomal RNA gene sequences. J Parasitol 1999;85:77-83.
18. Eberhard ML, Pieniazek NJ, Arrowood MJ. Laboratory diagnosis of *Cyclospora* infections. Arch Pathol Lab Med 1997;121:792-7.
19. Visvesvara GS, Moura H, Kovacs-Nace E, Wallace S, Eberhard ML. Uniform staining of *Cyclospora* oocysts in fecal smears by a modified safranin technique with microwave heating. J Clin Microbiol 1997;35:730-3.
20. Thompson JD, Higgins DG, Gibson TJ. CLUSTAL W: improving the sensitivity of progressive multiple sequence alignment through sequence weighting, position specific gap penalties and weight matrix choice. Nucleic Acids Research 1994;22:4673-80.
21. Strimmer K, von Haeseler A. Quartet puzzling: a quartet maximum likelihood method for reconstructing tree topologies. Mol Biol Evol 1996;13:964-9.
22. Swofford DL. PAUP*: Phylogenetic analysis using parsimony (and other methods). 1998. Ver 4.0b1. Sunderland (MA): Sinauer Associates.

## References

[PDF_00581] Ford P. L., Duszynski D. W. (1989). Coccidian parasites (Apicomplexa: Eimeriidae) from insectivores. VII. Six new species from the hairy-tailed mole, Parascalops breweri.. J Parasitol.

[PDF_00576] Ford P. L., Duszynski D. W., McAllister C. T. (1990). Coccidia (Apicomplexa) from heteromyid rodents in the southwestern United States, Baja California, and northern Mexico with three new species from Chaetodipus hispidus.. J Parasitol.

[PDF_00556] García-López H. L., Rodríguez-Tovar L. E., Medina-De la Garza C. E. (1996). Identification of Cyclospora in poultry.. Emerg Infect Dis.

[PDF_00545] Herwaldt B. L., Ackers M. L. (1997). An outbreak in 1996 of cyclosporiasis associated with imported raspberries. The Cyclospora Working Group.. N Engl J Med.

[PDF_00540] Koumans E. H., Katz D. J., Malecki J. M., Kumar S., Wahlquist S. P., Arrowood M. J., Hightower A. W., Herwaldt B. L. (1998). An outbreak of cyclosporiasis in Florida in 1995: a harbinger of multistate outbreaks in 1996 and 1997.. Am J Trop Med Hyg.

[PDF_00537] Ortega Y. R., Gilman R. H., Sterling C. R. (1994). A new coccidian parasite (Apicomplexa: Eimeriidae) from humans.. J Parasitol.

[PDF_00585] Ortega Y. R., Nagle R., Gilman R. H., Watanabe J., Miyagui J., Quispe H., Kanagusuku P., Roxas C., Sterling C. R. (1997). Pathologic and clinical findings in patients with cyclosporiasis and a description of intracellular parasite life-cycle stages.. J Infect Dis.

[PDF_00553] Pieniazek N. J., Herwaldt B. L. (1997). Reevaluating the molecular taxonomy: is human-associated Cyclospora a mammalian Eimeria species?. Emerg Infect Dis.

[PDF_00573] Pieniazek N. J., Slemenda S. B., da Silva A. J., Alfano E. M., Arrowood M. J. (1996). PCR confirmation of infection with Cyclospora cayetanensis.. Emerg Infect Dis.

[PDF_00548] Relman D. A., Schmidt T. M., Gajadhar A., Sogin M., Cross J., Yoder K., Sethabutr O., Echeverria P. (1996). Molecular phylogenetic analysis of Cyclospora, the human intestinal pathogen, suggests that it is closely related to Eimeria species.. J Infect Dis.

[PDF_00566] Smith H. V., Paton C. A., Girdwood R. W., Mtambo M. M. (1996). Cyclospora in non-human primates in Gombe, Tanzania.. Vet Rec.

[PDF_00593] Sterling C. R., Ortega Y. R. (1999). Cyclospora: an enigma worth unraveling.. Emerg Infect Dis.

[PDF_00606] Thompson J. D., Higgins D. G., Gibson T. J. (1994). CLUSTAL W: improving the sensitivity of progressive multiple sequence alignment through sequence weighting, position-specific gap penalties and weight matrix choice.. Nucleic Acids Res.

[PDF_00562] Yai L. E., Bauab A. R., Hirschfeld M. P., de Oliveira M. L., Damaceno J. T. (1997). The first two cases of Cyclospora in dogs, São Paulo, Brazil.. Rev Inst Med Trop Sao Paulo.

[PDF_00559] Zerpa R., Uchima N., Huicho L. (1995). Cyclospora cayetanensis associated with watery diarrhoea in Peruvian patients.. J Trop Med Hyg.

[PDF_00569] da Silva A. J., Bornay-Llinares F. J., Moura I. N., Slemenda S. B., Tuttle J. L., Pieniazek N. J. (1999). Fast and reliable extraction of protozoan parasite DNA from fecal specimens.. Mol Diagn.

